# Private sector participation in delivering tertiary health care: a dichotomy of access and affordability across two Indian states

**DOI:** 10.1093/heapol/czu061

**Published:** 2015-03-09

**Authors:** Anuradha Katyal, Prabal Vikram Singh, Sofi Bergkvist, Amit Samarth, Mala Rao

**Affiliations:** ^1^ACCESS Health International, Center for Emerging Market Solutions, Indian School of Business, Gachibowli, Hyderabad 500032, India, ^2^Oxford Policy Management, Central Delhi, Delhi, India, ^3^SughaVazhvu Healthcare, A2 L.P Amsavalli Illam, Arulananda Nagar, Thanjavur 613007, India and ^4^University of East London, Institute for Health and Human Development (IHHD), UH250, Stratford Campus, University of East London, Water Lane, London E15 4LZ, UK

**Keywords:** Health financing, health insurance, health policy, public and private sector

## Abstract

Poor quality care in public sector hospitals coupled with the costs of care in the private sector have trapped India's poor in a vicious cycle of poverty, ill health and debt for many decades. To address this, the governments of Andhra Pradesh (AP) and Maharashtra (MH), India, have attempted to improve people’s access to hospital care by partnering with the private sector. A number of government-sponsored schemes with differing specifications have been launched to facilitate this strategy.

**Aims** This article aims to compare changes in access to, and affordability and efficiency of private and public hospital inpatient (IP) treatments between MH and AP from 2004 to 2012 and to assess whether the health financing innovations in one state resulted in larger or smaller benefits compared with the other.

**Methods** We used data from household surveys conducted in 2004 and 2012 in the two states and undertook a difference-in-difference (DID) analysis. The results focus on hospitalization, out-of-pocket expenditure and length of stay.

**Results** The average IP expenditure for private hospital care has increased in both states, but more so in MH. There was also an observable increase in both utilization of and expenditure on nephrology treatment in private hospitals in AP. The duration of stay recorded in days for private hospitals has increased slightly in MH and declined in AP with a significant DID. The utilization of public hospitals has reduced in AP and increased in MH.

**Conclusion** The state of AP appears to have benefited more than MH in terms of improved access to care by involving the private sector. The Aarogyasri scheme is likely to have contributed to these impacts in AP at least in part. Our study needs to be followed up with repeated evaluations to ascertain the long-term impacts of involving the private sector in providing hospital care.

**KEY MESSAGES**

- The findings of the study highlight the fact that participation of private sector in the Rajiv Aarogyasri scheme in Andhra Pradesh has improved the access to health care in the state.
- Despite the improvement in access to healthcare there is no evidence to support the fact that a huge volume of patients have been driven to seek healthcare in private hospitals especially among the unreached rural population.
- It has to a certain extent mitigated the cost of healthcare in the state especially when compared to the control state in the study.

## Introduction

We stand at a moment of exceptional possibility. A moment when global health and development goals that long seemed unattainable have moved within our reach.

World Bank Group President Jim Yong Kim’s Speech at World Health Assembly made on 21 May 2013

The Indian healthcare system has witnessed many changes in the last decade. There has been an improvement in the health indices such as infant mortality, maternal mortality and life expectancy (CESS 2012). Though India is some distance away from achieving some of its Millennium Development Goals (MDG targets), much progress has been made towards these goals. There has been a slew of reforms in the health sector beginning with the launch of the National Rural Health Mission in 2005 with an aim to *‘*improve the availability of and access to quality health care by people, especially for those residing in rural areas, the poor, women and children’ (NRHM 2005).

Despite these efforts, there is a huge cross section of the population that continues to struggle to gain access to affordable good quality healthcare. Although the rich can access healthcare by paying large sums of money, the poor are under major threat of financial duress, sometimes following a single episode of illness that may push even the middle income groups into poverty or indebtedness (Rao *et al.* 2011). Although the government-funded facilities struggle to provide services to the vast and growing population, the exponential growth of private facilities has been highly unregulated and unchecked. Meanwhile, low levels of public health financing, supply side gaps, an acute shortage of human resources and the rising cost of healthcare continue to severely affect access, affordability and quality of health services across the country.

Against this background, the government has been attempting to address two main challenges: to ensure that all citizens can access healthcare equitably and to ensure that healthcare is made available at an affordable cost and without compromising on quality.

To achieve this, there have been attempts to facilitate access to the state-of-the-art private hospitals for the benefit of the ‘unreached and underprivileged’. During the past two decades, central and state governments have designed a number of different state-funded insurance schemes aimed at increasing access to healthcare and making hospitalization affordable for the poor. One of the first new generation schemes to be launched was the Rajiv Aarogyasri Scheme (RAS) developed and funded by the Government of Andhra Pradesh (AP) in 2007 (Fan *et al.* 2012). The scheme provides free access to over 900 secondary and tertiary procedures and covers more than 75% of the population (RAS 2012). The Rashtriya Swasthya Bima Yojana (RSBY) was another scheme that was launched nationally in 2008 (RSBY 2013a). This scheme is jointly funded by the central and state governments. In Maharashtra (MH) enrolment to RSBY began in mid-2009, whereas in AP enrolment began in 2013 and has only occurred in one district (RSBY 2013b). Both the schemes ‘empanel’ private and public hospitals to provide treatments funded by them.

## Role of the private health sector in India

**Figure 1:**
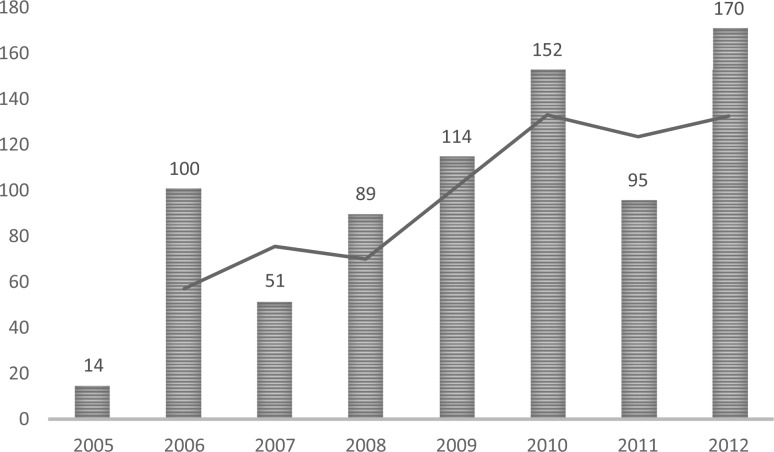
The number of private hospitals receiving recognition as teaching hospitals per year by the Department of Medical Education in AP (APDME 2012).

In the early 1950s, after the independence of India, the private sector constituted only 8% of the market (Venkat Raman 2008). By contrast, according to the National Health Accounts 2009, the share of private expenditure was 73% of the total health expenditure (Planning Commission of India 2013). In six and a half decades, the private sector share has grown nine times and the public sector has declined in absolute terms by a third. The Indian healthcare market was expected to grow by an estimated Unites States Dollar (USD) 40 billion by 2012 (PwC 2007) while the private health sector market (in terms of the amount spent on healthcare as a private industry) was valued at around USD 29 billion in 2009 (PwC 2007). Meanwhile, public expenditure on health has hovered at around 1% of gross domestic product (GDP). The private health sector contributes around 70% of all the hospitals and ∼40% of total hospital beds (PwC 2007). In RSBY, 70% of the hospitals enrolled are private (La Forgia and Nagpal 2012), and in the RAS private hospitals make up around 78% of the providers (RAS 2012). According to a study of government-sponsored health insurance schemes in India (Singh and Kalvakuntla 2013), the introduction of RAS in 2007 resulted in a substantial increase in utilization of both public and private facilities, but as the scheme grew, the utilization of private facilities kept increasing while that of public facilities stabilized. According to the Department of Medical Education in AP, there has been a steady increase in the number of private hospitals gaining formal ‘recognition’ as teaching hospitals after the introduction of RAS, as illustrated in Figure 1.

The evidence illustrates that the private health sector has a key role to play in delivering health services in AP and that its role is likely to remain important in the future. Consequently, it is important to understand how the private sector facilities contribute to health care access, affordability and quality (Mallipeddi *et al.* 2009).

In AP following the launch of the RAS, there have also been attempts to strengthen public hospitals, which were in a state of neglect, leaving the poor no option but to access costly private facilities (Nagulapalli and Rokkam 2013). The government has been providing financial incentives to the surgical teams of public hospitals for every treatment provided under the umbrella of the RAS (Niloufer Hospital 2014). In parallel, the participation of private healthcare providers was intended to encourage the public sector to match the quality of their services by inducing competition. Furthermore, the government of AP and the Aarogyasri Healthcare Trust (the organization which commissions the RAS) have encouraged private–public partnerships to improve care. For example, they have supported the development of technologically advanced dialysis units in public hospitals in partnership with a private company (B. Braun) to address the high unmet needs of patients with renal disease (B. Braun 2013).

### Aims of the study

Against this background, this article aims to assess changes in accessibility, affordability and perceptions of efficiency of private health care inpatient (IP) treatment across the states of MH and AP from 2004–05 to 2012. In our study, we compared two states with relatively similar economic and demographic conditions (Table 1).

**Table 1:** Urban and rural populations and households surveyed in 2004 and 2012 in AP and MH^a^

	AP		MH	
	2004	2012	2004	2012
Population in the entire state	76 210 007^b^	84 665 533^c^	96 878 627^b^	112 372 972^c^
Urban population in the state	20 808 940^b^	28 353 745^c^	41 100 980^b^	50 827 531^c^
Rural population in the state	55 401 067^b^	56 311 788^c^	55 777 647^b^	61 545 441^c^
Total households in the state (urban)	4 397 138^b^	6 778 225^c^	8 403 224^b^	10 813 928^c^
Total households in the state (rural)	12 607 167^b^	14 246 309^c^	11 173 512^b^	13 016 652^c^
Total households in the state	17 004 305^b^	21 024 534^c^	19 576 736^b^	23 830 580^c^
FSUs surveyed (urban)	183^d^	372^e^	267^d^	504^e^
FSUs surveyed (rural)	325^d^	491^e^	265^d^	504^e^
Total households surveyed (urban)	1824^d^	3715^e^	2664^d^	5038^e^
Total households surveyed (rural)	3235^d^	4908^e^	2650^d^	5035^e^

^a^FSU, first-stage unit.

^b^2001 census.

^c^2011 census.

^d^NSSO 2004.

^e^The NSSO 66th round had 492 rural FSUs in AP, but 1 FSU was found to be uninhabited. The list of FSUs which were surveyed in the 66th round were obtained from the Coordination and Publication Division of the National Survey Sample Organization after the investigators requested deputy director general to instruct their regional offices to provide these.

## Methodology

We used a retrospective, longitudinal, controlled quasi-experimental study to compare IP health care-related expenditures and behaviours (HREB) in AP (the state implementing RAS) and in MH, the state implementing RSBY (Angrist and Pischke 2009). HREBs were measured in both AP and MH by two waves of household surveys before (2004) and after (2012) the introduction of RAS and RSBY.

### Baseline data

We used unit-level data from the National Sample Survey Organization (NSSO) 60th round survey, conducted in 2004. This decennial survey was the most recent round measuring population morbidity profiles, use of health care services including hospitalized and non-hospitalized treatments and expenditures incurred (NSSO 2004). The household survey used a multi-stage stratified sampling methodology (NSSO 2004), to identify a representative random population sample and an interviewer completed a questionnaire to obtain measures of HREB along with sociodemographic, household expenditure and other information (See Supplementary information, Annex 1).

### Follow-up survey: 2012

We used the same household survey design and methods to collect post-intervention data in AP and MH as those used by NSSO. Briefly, the household survey used a multi-stage stratified sampling methodology with the ‘first-stage units’ (FSUs) identical to those used by NSSO in their 66th round (2008–09), the latest round for which FSUs had been mapped. However, the FSUs were not the same as those in NSSO 2004, our baseline survey, rapid urbanization having changed substantially the urban–rural landscape of both states and thus the geographical basis for sampling units.

## Analysis

The key to infer causality to a particular intervention lies in identifying the confounders and assessing the trends in the behaviour of the control and the treatment group (Angrist and Pischke 2009). But, in the absence of multidimensional or panel data our best approach for this study was to use baseline (2004) and endline (2012 our study) data for these two states and analyse the data using a difference-in-difference (DID) methodology.

The DID of outcome ($Y_{\text{DD}})$ is

$$\left(Y_{2012}^{\text{AP}}-\;Y_{2004}^{\text{AP}})-(Y_{2012}^{\text{MH}}-\;Y_{2004}^{\text{MH}}\right)$$

where the scripts for *Y* refer to the respective states and the years when the surveys were done. Confidence intervals were calculated from the standard error $Y_{\text{DD}}$ and the *P* value for the null-hypothesis ($Y_{\text{DD}}=0$) was tested using the Wald test as $\;t=\frac{Y_{\text{DD}}}{{\text{SE}}_{Y_{\text{DD}}}}$ with one degree of freedom. $Y_{\text{DD}}$ was estimated using ordinary least-square regression

$$y_{it}=\;\beta_{0}+\beta_{1}{\text{state}}_{i}+\beta_{\text{2}}{\text{survey}}_{t}+\beta_{3}{\left(\text{state}*\text{survey}\right)}_{it}+\overset{m}{\underset{k=1}{\sum }}\beta_{3+k}{\text{covariate}}_{k}+\varepsilon$$

where $y_{it}$ is the outcome, state is a dummy variable with 0 for MH and 1 for AP and survey is a dummy variable with 0 for the 2004 survey and 1 for the 2012 survey. The coefficient for the interaction term, $\beta_{3}$, gives the DID estimate, $Y_{\text{DD}}$, while ε*_i_* is an idiosyncratic error term. Robust standard errors of $Y_{\text{DD}}$ were calculated to account for design effect due to clustering of households within FSUs using Stata survey commands. A positive value for $Y_{\text{DD}}$ suggested that the change in the outcome in AP was more than the change in MH and a negative value would suggest the reverse. NSSO provided weights along with the unit-level data for the 2004 data, and we developed weights using the same method for our survey.

The basic DID results were obtained using the above regression with covariates excluded. The adjusted DID results are obtained using the above regression with *m* = 9 covariates, namely the gender of head of household, a dummy variable capturing whether the household lives in a rural or urban location, three dummy variables capturing the household’s social group (the lowest is the excluded category), and four asset quintile dummies (the bottom is the excluded category). This regression simply compares the change between 2004 and 2012 between the two states: the coefficient *β*_1_ gives the extra growth in *y* in AP over and above that in MH. If Aarogyasri and other initiatives implemented between 2004 and 2012 in AP had the same effect on *y* as those initiatives implemented between 2004 and 2012 in MH, *β*_1_ will be zero. The $\beta_{3}$ will reduce any bias in our estimate due to a correlation between the $\beta_{3}$ and the AP dummy, and will also give us greater precision in our estimate of *β*_0_ (Angrist and Pischke 2009).

## Variables of interest

### Access to IP care

Hospitalization rate: This was estimated as the numbers of individuals hospitalized during the previous year per 1000 population. In addition, among those hospitalized, the utilization of public and private hospitals overall and for cardiac and nephrology has been analysed using DID. These values are a proportion of those being hospitalized.

### Cost

Expenditure on hospitalization: the average out-of-pocket expenditure (OOPE) for IP care per individual within 1 year of the survey was estimated for the population from questionnaire responses for AP and MH from both baseline and endline data.

Expenditure on ‘high-cost’ treatments: the average OOPE for IP care within 1 year of the survey was estimated for both public and private hospitals per episode of cardiac and nephrology treatments, which were used as proxies for high-cost treatments. These procedures are some of the most expensive and they also require long-term follow-up treatments.

### Efficiency

The duration of hospital stay has been used as a proxy for efficiency. This variable is self-reported and measured in number of days of stay in the hospital. Data have been analysed by rural and urban residence.

## Results

For the various variables of interest and the sub groups, the results are shown as the averages in the baseline, the change in the 2012 survey when compared with the baseline, with the DID estimate with 95% confidence interval and the respective *P* values of the DID comparing AP with MH.

### Access to IP care

Table 2 shows the proportion of IP cases in various subgroups among those hospitalized. In general, utilization of private hospitals has increased in AP and decreased in MH. The likelihood of admission to a private hospital was significant for hospitalizations among urban households (*P* = 0.0002) and in particular for nephrology treatment among urban households (*P* = 0.0007).

**Table 2:** Change in proportion of IP cases in public and private hospitals (among those hospitalized)

	IP cases	Baseline mean (95% CI)		Change 2004:2012 mean (95% CI)		DID estimate	
		MH	AP	MH	AP	Mean (95% CI)	*P*
Private	Overall	0.72 (0.7:0.73)	0.7 (0.69:0.72)	−0.011 (−0.053:0.031)	0.065 (0.018:0.11)	0.076 (−0.012:0.14)	0.02

						DID estimate with covariates	
						Mean (95% CI)	*P*
	
						0.05 (−0.007:0.11)	0.03
	Rural	0.72 (0.69:0.75)	0.73 (0.7:0.75)	0.030 (−0.027:0.089)	0.028 (−0.023:0.081)	−0.0019 (−0.080:0.076)	0.96
	Urban	0.72 (0.70:0.75)	0.63 (0.6:0.66)	−0.067 (−0.13:−0.0063)	0.14 (0.047:0.23)	0.21 (0.095:0.31)	0.0002
	Cardiac	0.056 (0.037:0.076)	0.072 (0.038:0.1)	−0.017 (−.038:0.0038)	−0.018 (−0.05:0.015)	−0.0015 (−0.042:0.039)	0.94
	Cardiac rural	0.037 (0.017:0.057)	0.06 (0.016:0.1)	−0.0056 (−0.028:0.016)	−0.008 (−0.055:0.037)	−0.0031 (−0.054:0.048)	0.9
	Cardiac urban	0.08 (0.046:0.011)	0.097 (0.057:0.13)	−0.028 (−0.06:0.0085)	−0.04 (−0.083:0.002)	−0.012 (−0.069:0.043)	0.65
	Nephrology	0.035 (0.047:0.09)	0.069 (0.047:0.09)	−0.027 (−0.051:−0.004)	0.0023 (−.012:0.021)	0.029 (−0.0036:0.06)	0.053
	Nephrology rural	0.052 (0.023:0.08)	0.042 (0.019:0.065)	−0.009 (−0.026:0.02)	−0.006 (−0.032:0.02)	0.0036 (−0.037:0.04)	0.86
	Nephrology urban	0.088 (0.055:0.12)	0.018 (0.005:0.031)	−0.04 (−0.084:0.014)	0.021 (0.0009:0.041)	0.07 (0.03:0.11)	0.0007

Public	Overall	0.27 (0.23:0.31)	0.3 (0.26:0.34)	0.011 (−0.032:0.053)	−0.064 (−0.11:−0.017)	−0.075 (−0.14:0.0125)	0.019

						DID estimate with covariates	
						Mean (95% CI)	*P*
	
						−0.06 (−0.11:0.005)	0.074
	Rural	0.28 (0.21:0.35)	0.27 (0.23:0.31)	−0.03 (−0.09:0.028)	−0.028 (−0.08:0.02)	0.0019 (−0.076:0.08)	0.96
	Urban	0.26 (0.21:0.32)	0.36 (0.28:0.45)	0.067 (−.062:0.12)	−0.14 (−0.23:−0.047)	−0.2 (−0.31:−0.095)	0.0002
	Cardiac	0.0034 (0.02:0.049)	0.045 (0.025:0.065)	0.005 (−0.015:0.025)	−0.014 (−0.038:0.11)	−0.019 (−0.05:0.013)	0.25
	Cardiac rural	0.005 (−0.00034:0.011)	0.042 (0.014:0.07)	−0.014 (−0.012:0.04)	−0.021 (−0.053:0.0098)	−0.036 (−0.076:0.00513)	0.089
	Cardiac urban	0.053 (0.026:0.078)	0.05 (0.02:0.0786)	−0.008 (−0.04:0.024)	0.011 (−0.031:0.054)	0.019 (−0.034:0.072)	0.48
	Nephrology	0.048 (0.024:0.070)	0.039 (0.0083:0.069)	−0.026 (−0.05:−0.0016)	−0.012 (−0.046:0.02)	0.014 (−0.028:0.055)	0.52
	Nephrology rural	0.03 (0.012:0.049)	0.0078 (0.0017:0.0014)	−0.014 (−0.035:0.0076)	0.018 (−0.0029:0.036)	0.031 (0.0034:0.059)	0.028
	Nephrology urban	0.069 (0.023:0.11)	0.083 (0.011:0.16)	−0.043 (−0.09:0.0042)	−0.055 (−0.13:−0.019)	−0.012 (−0.1:0.077)	0.79

CI, confidence intervals.

The pattern of utilization of public hospitals was different. The overall utilization of public facilities has reduced in both the states and more so in AP (*P* = 0.087). There was an increase in utilization of public facilities in MH and a reduction in AP for urban households (*P* = 0.002) and cardiac hospitalizations in rural households (*P*-value 0.089). An opposite trend was observed for nephrology care among rural households (*P* = 0.028).

### Changes in average IP expenditure—public vs private

Table 3 shows averages of IP expenditure among those hospitalized in private and public healthcare facilities in 2004 and 2012. The table also shows the real terms change (deflated to 2004 prices) in these outcomes at follow-up and the DID estimate comparing AP with MH.

**Table 3:** Average expenditure among those hospitalized in the state (all values in Indian National Rupee (INR) deflated to 2004)

	IP cases	Baseline mean (95% CI)		Change 2004:2012 mean (95% CI)		DID estimate	
		MH	AP	MH	AP	Mean (95% CI)	*P*
Private	Overall	5718.1 (5118.6:6317.6)	5758.8 (4193.8:7323.9)	3435.5 (2521.7:4349.3)	1358.9 (−330.1:3048)	−2076.5 (−3996:−157)	0.04

						DID estimate with covariates	
						Mean (95% CI)	*P*
	
						−2306.9 (−4203:−410.7)	0.017
	Rural	6274.3 (5547.4:7001.2)	6545.2 (5766.6:7323.9)	3397.9 (2096.8:4699)	1271.4 (108.8:2434.024	−1620.6 (−3052:−189.3)	0.026
	Urban	9554.7 (8059.4:11050)	11804.8 (5791.04:17818.5)	2892.1 (832.9:4951.5)	−2902 (−9118.2:314.2)	−3235.3 (−8006.4:1535.9)	0.18
	Cardiac	1065.2 (618.3:1512.1)	1067.6 (617.2:1518)	143.3 (−472.8:759.3)	−228.7 (−737.2:279.8)	−371.9 (−1170.4:426.5)	0.36
	Cardiac rural	227.1 (109:345.3)	895.9 (419.3:1372.6)	337.7 (−51.04:726.53)	−206.0 (−747.04:355.04)	−543.7 (−1209.6:122.2)	0.11
	Cardiac urban	2076.8 (1107.2:3046.3)	1453.9 (472.5106:2435.371)	147.6 (−1179.4:1474.7)	−259.5 (−1370.6:851.6)	−407.1 (−2137.1:1322.9)	0.64
	Nephrology	814.1 (542.2:1086.01)	292.3 (155.8:428.9)	−220.1 (−550.03:109.9)	413.4 (−8.2:835.1)	633.5 (98.3:1168.67)	0.19
	Nephrology rural	479.3 (248.5:710.1)	364 (183.085:544.82)	77.09 (−263.5:417.7)	383.31 (−194.1:960.7)	306.2 (−363.8:976.2)	0.37
	Nephrology urban	1217.7 (685.1:1750.3)	131.2 (−43.8:306.3)	−564.2 (−1165.7:37.4)	475.5 (101.8:849.2)	1039.7 (331.8:1747.6)	0.004

Public	Overall	1440.4 (1096:1785.005)	1010.1 (772.3:1247.9)	7713.1 (6944.7:8481.4)	6107.7 (5431.3:6784.2)	−1605.3 (−2628.6:−582.1)	0.002

						DID estimate with covariates	
						Mean (95%CI)	*P*
	
						−1711 (−2776.1:−647.6)	0.002
	Rural	2223.5 (1172.1:3274.8)	1936 (1460:2412.0)	1897.1 (241.3:3553)	2212 (677.5:3746.5)	−833.7 (−2100.8:433.5)	0.2
	Urban	2162.7 (1449.0: 2876.4)	1359.9 (855.9:1863.9)	3957.4 (2428.6:5486.2)	1697.3 (780.1:2614.5)	−2585.4 (−4433.9:−736.9)	0.0061
	Cardiac	315.7 (57:574.5)	201.8 (23.9:379.6)	333.5 (−235.7:902.6)	−18.8 (−226.1:188.6)	−352.2 (−957.7253.2777)	0.2541
	Cardiac rural	331.4 (−80.7:743.1)	211.5 (−66.3:489.3)	209.5 (−733.7:1152.7)	−70.6 (−367:225.7009)	−384.5 (−868.0:99)	0.12
	Cardiac urban	295.7 (29.92:561.4)	187.6 (21:354.2)	467.6 (−125.3:1060.4)	106.9 (−218.9:432.7)	−360.6 (−1036.6:315.4)	0.2956
	Nephrology	84.7 (7.5:161.9)	104.2 (50.5:158)	55.0 (−76.4:186.4)	221.3 (−120.4:563.1)	−166.3 (−532.3:199.7)	0.37
	Nephrology rural	58.4 (2.4:114.4)	24.7 (−1.6:51.0)	22.3 (−66.5:111.0)	110.8 (−30.2:251.9)	88.6 (−78:55.1)	0.3
	Nephrology urban	163.1 (65.1:261.1)	171.7 (−10.4:353.9)	418.2 (−276.1:1112.5)	−20.8 (−243.4:201.8)	−439.0 (−1167.7:289.6)	0.24

The overall expenditure on IP care per episode in private facilities has increased in both states (more so in MH, *P* = 0.04). Expenditures on high-cost treatments such as cardiac care and nephrology show a mixed picture. The expenditure on nephrology hospitalizations in private facilities has increased in urban households (*P* = 0.004). The average expenditure on public facilities has also increased in both states and more again in MH (*P* = 0002). A similar trend is observed in rural and urban households. The expenditure on cardiac care in public hospitals has reduced in AP while it has increased in MH. The expenditure on nephrology has increased in both states but more so in AP.

### Efficiency

There has been a minor increase in the average length of stay (recorded in days) in private hospitals in MH while we found a decrease of ∼33% in average length of stay in private hospitals in. The results from the DID analysis gave an average reduction of 3.2 days in AP (*P* = 0.002) and among those in rural households (*P* = 0.007). For public hospitals, it has decreased in both AP and MH, and significantly more so, with an average of 4.2 days, in AP for rural households (*P* = 0.09), as shown in Table 4.

**Table 4:** The duration of hospital stay in days

	IP cases	Baseline mean (95% CI)		Change 2004:2012 mean (95% CI)		DID estimate	
		MH	AP	MH	AP	Mean (95% CI)	*P*
Private	Overall	6.6 (6.1:7.2)	10 (8.1:11.8)	0.18 (−0.46:0.81)	−3 (−4.9:−1.2)	−3.2 (−5.3:−1.2)	0.002

						DID estimate with covariates	
						Mean (95% CI)	*P*
	
						−3.2 (−5.4:−1.2)	0.003
	Rural	6.9 (6.2:7.7)	10.5 (8.1:12.9)	−0.1 (−1:0.79)	−3.8 (−2.9:−0.96)	−3.7 (−6.3:−1)	0.007
	Urban	6.2 (5.4:7)	8.9 (6.5:11.2)	0.5 (−4:1.4)	−1.3 (−3.7:−1.2)	−1.8 (−4.4:0.8)	0.17

Public	Overall	9.7 (7.4:11.9)	11.5 (10.1:12.9)	−2.5 (−4.9:−0.05)	−4.5 (−6.3:−2.6)	−2 (−5.1:1.1)	0.2

						DID estimate with covariates	
						Mean (95% CI)	*P*
	
						−2 (−5.0:1.1)	0.2
	Rural	10.1 (6.4:14)	13.3 (11.2:15.3)	−1.9 (−6:2.1)	−6 (−8.7:−3.5)	−4.2 (−9:0.6)	0.09
	Urban	9.3 (7.5:11)	8.9 (7.7:10)	−3 (−4.9:−0.9)	−2 (−3.8:−0.5)	0.7 (−1.8:3.2)	0.59

## Limitations

### 

Despite the states being similar in their profiles, there may have been factors resulting in unobserved changes between the two populations. These factors may have driven the direction of the DID. The DID analysis itself assumes that there has been not much time variance in the subjects under study which is not true (Angrist and Pischke 2009). The 2004 NSSO survey which served as our baseline survey was conducted between January and June 2004 (NSSO 2004). Our 2012 survey was carried out over a period of 3 months from June to September. The morbidity and mortality patterns recorded in different time periods may vary and could have influenced the data.

## Discussion and policy implications

The utilization of private facilities in AP shows significant difference in facilities for certain treatments; this could be explained by the presence of the Aarogyasri scheme which provides access to private facilities. In the earlier sections, it has also been highlighted that there was an increase in recognition of private teaching hospitals after the launch of the RAS; this could have further influenced the utilization of private hospitals. The scheme not only provides financial protection, it also gives the beneficiaries more choice of providers including private hospitals for specified conditions. Because the Aarogyasri beneficiaries are entitled to additional funding for nephrology treatments the utilization may have increased in the state. Even though the findings are in general skewed in favour of utilization of private hospitals, the increased utilization of public hospitals among rural households for nephrology treatments may reflect greater use of state-of-the-art dialysis units developed in public hospitals by the public–private partnership between B. Braun, Government of AP and the Aarogyasri Healthcare Trust as mentioned earlier. The rural patients may have travelled to the nearest public hospitals with dialysis facilities, these being distributed across several cities of AP (B.Braun 2013). These findings suggest that the participation of private health care providers in partnership with government may have resulted in improved access to healthcare. Our findings may suggest that the positive effects of Aarogyasri detected by other studies at an early stage of the roll-out of the scheme have been sustained. Automatic enrolment into the scheme, near universality of coverage and no requirement for enrollee contributions may have contributed further to the significant DIDs (Fan *et al.* 2012).

Unlike nephrology, the utilization of cardiac care has decreased in both public and private hospitals in AP. This is consistent with the utilization patterns of the scheme itself. When Aarogyasri was launched in 2007, ∼52% of the surgeries carried out were for cardiology and cardiothoracic surgeries (RAS 2013). However these figures declined to 25% in 2008, 16% in 2009, 14% in 2010 and 12% in 2011, 2012 and 2013. This trend may indicate that as the scheme was being rolled out there was a huge unmet need for cardiac surgery, which was addressed by the Aarogyasri scheme in its post-launch phase.

Another possible explanation for comparatively greater utilization of private facilities in AP than in MH is that only 2 million households were enrolled under RSBY (RSBY 2013a) in MH by the time of the survey, while in AP more than 70 million (RAS 2012) families were enrolled under RAS, i.e. more than 80% of the population of AP. Studies have also reported that the utilization of RSBY has been low in MH (Thakur and Ghosh 2013) especially when compared with the other states in which it has been launched (Palacios *et al.* 2011). The ‘Critical Assessment of the Existing Health Insurance Models in India’ by Reddy *et al.* (2011) has highlighted that while only 12% of MH’s population is covered by health insurance, in AP the coverage is as high as 87% (near universal). An assessment of a community-based health insurance scheme in the neighbouring state of Karnataka also demonstrated an increased utilization of private facilities for surgeries (Agarwal 2010). Furthermore, a descriptive study of the Aarogyasri scheme in its early years highlighted that, given a choice, the poor prefer clean hospitals with polite staff, predominantly available in the private sector (Rao *et al.* 2011). A notable observation is that admissions to private hospitals among rural households have increased in both MH and AP. This may be a result of an increase in private hospitals in smaller towns which are in the vicinity of the rural areas and also the availability of better ambulance facilities.

With increase in utilization, average OOPE has also increased in both public and private facilities for both the states, but more so in MH. The expenditure on cardiac care in private hospitals has reduced in AP in both rural and urban areas, and increased in MH, although the DID is not significant. The expenditure on cardiac care in public hospitals has reduced in AP while it has increased in MH and that on nephrology has increased in both states but more so in AP, even though none of these results are statistically significant. The explanation may be that, although the scheme covers not only the treatment but also additional costs such as for food and transport, patients needing nephrology treatments which require frequent hospitalization, and their families unaware of these benefits, may be bearing these additional costs. Nephrology treatments also need long-term care and medication; perhaps the patients are unaware of the fact that they are entitled to 7 months of follow-up medicine in addition to the other benefits. The expenditure on hospitalizations for cardiac care in public hospitals has increased in MH and reduced in AP, despite decreased utilization in both states.

In AP, there has been a reduction in average length of hospital stay in comparison with MH. This change may be directly related to the increased numbers of those seeking hospital care, and shorter durations of admission in response to fixed treatment costs reimbursed by the RAS. Given their entitlement to hospital care under the RAS in AP, people may have begun seeking care at an earlier stage and also for less serious complaints, because the treatment is offered free of cost.

## Conclusion

The findings of this study illustrate that providing a scheme such as the Aarogyasri, which involves the private sector, not only benefits those covered under the scheme but also indirectly motivates the healthcare providers to establish better facilities in even smaller towns, hence improving access to hospital care for serious illness. The Aarogyasri scheme may also be influencing greater efficiency of care in both public and private hospitals. The fact that competition is encouraged between public and private hospitals and public hospital staff are incentivized to improve their services, may result in improved morale and quality of care and services in public hospitals as well as improved ethics and behaviours among the private hospitals, mindful of the increasing competition from the public sector hospitals. The Aarogyasri scheme has also demonstrated an important additional benefit of public–private partnerships; that public providers may be enabled to provide technologically advanced treatments in state-of-the-art facilities developed in their hospitals. This study assessed the impact of private hospital participation in the Aarogyasri scheme, by exploring changes in access to and household expenditure on IP care. AP appears to have benefited more than MH in terms of improving access to care over time. This positive change is likely to be attributable to the RAS, which encourages the involvement of the private sector in care provision, at least in part.

Other states have replicated Aarogyasri and there are opportunities to introduce changes that can improve care not only in AP but also influence the programmes of other states. It is now important to look closer at the quality of services to make sure that the increase in use of services has not compromised the quality. Additional studies such as facility surveys and clinical audits (in addition to those carried out by the government itself) need to be undertaken to compare the quality of care provided by the private and public hospitals, and to understand further, the long-term impact of private participation in providing hospital care under the aegis of a publicly funded health financing scheme.

## Supplementary Data

Supplementary data are available at *HEAPOL* online.

Supplementary Data
