# Supplementary material for: Private sector participation in delivering tertiary health care: a dichotomy of access and affordability across two Indian states
Source: Health Policy Plan. 2015 Mar 9;30(Suppl 1):i23–31. doi: 10.1093/heapol/czu061 (PMC4353890; doi:10.1093/heapol/czu061)
Supplement: Supplementary Data [file supp_30_suppl-1_i23__index.html]

Private sector participation in delivering tertiary health care: a dichotomy of access and affordability across two Indian states — Supplementary Data 

# Private sector participation in delivering tertiary health care: a dichotomy of access and affordability across two Indian states

## Supplementary Data

files

**Files in this Data Supplement:**

- Supplementary Data - docx file
- Supplementary Data - docx file
- Supplementary Data - docx file
- Supplementary Data - docx file
- Supplementary Data - docx file
- Supplementary Data - docx file
- Supplementary Data - docx file
