# Supplementary material for: Private sector participation in delivering tertiary health care: a dichotomy of access and affordability across two Indian states
Source: Health Policy Plan. 2015 Mar 9;30(Suppl 1):i23–31. doi: 10.1093/heapol/czu061 (PMC4353890; doi:10.1093/heapol/czu061)
Supplement: Supplementary Data [file supp_czu061_Acronym_list.docx]

| AP | Andhra Pradesh |
| --- | --- |
| CESS | Centre for Economic and Social Studies |
| FSU | First Satge Units |
| GDP | Gross Domestic Product |
| HREB | Health Related Expenditures and Behaviours |
| IP | In-patient |
| MH | Maharashtra |
| NRHM | National Rural Health Mission |
| NSSO | National Sample Survey Organization |
| OOPE | Out of Pocket Expenditures |
| RAS | Rajiv Aarogyasri Scheme |
| RSBY | Rastriya Swasthya Bima Yojana |
| SSS | Second Stage Strata |
