# Supplementary material for: Private sector participation in delivering tertiary health care: a dichotomy of access and affordability across two Indian states
Source: Health Policy Plan. 2015 Mar 9;30(Suppl 1):i23–31. doi: 10.1093/heapol/czu061 (PMC4353890; doi:10.1093/heapol/czu061)
Supplement: Supplementary Data [file supp_czu061_Annex_on_sampling_methodology.docx]

# Annexure on Sampling and Data collection

The National Sample Survey Organization’s (NSSO) survey planning ensures equity of representation across the national socio-economic, demographic and geographic landscape. For each state or union territory, samples are divided into two sectors (rural and urban) in proportion to the provisional population as per the Census (NSSO, 2004).

The sampling methodology followed in our survey was that of the NSSO (healthcare, morbidity and condition of the aged) 60^th^ round, to ensure comparability with the baseline. However, the list of First Stage Units (FSUs) which were surveyed in the 66^th^ round were obtained from the Coordination and Publication Division of the National Survey Sample Organization after the investigators requested Deputy Director General to instruct its regional offices to provide these. A latest list of FSUs was used because rapid urbanization and population growth in the country has significantly changed the urban and rural landscape of the country since 2004.

We used a retrospective, longitudinal, controlled quasi-experimental study to compare inpatient health care related expenditures and behaviours (HREB) in AP (the state implementing the scheme) and in MH, the state implementing the Rashtriya Swasthya Bima Yojana(RSBY, the National Health Insurance Scheme) (Angrist & Pischke, 2009). HREBs were measured in both AP and MH by two waves of household surveys before (2004) and after (2012) the introduction of RAS and RSBY. The study protocol and questionnaire for the 2012 survey were reviewed and agreed by the Research Ethics Committee of the Administrative Staff College of India, Hyderabad. Informed signed consent was taken from the head of the household or a representative who agreed to provide information on the behalf of the adults and children in the family, prior to the administration of the questionnaire survey.

## First stage sampling

### Villages and urban blocks

A stratified multi-stage sampling method is used. The first stage units (FSUs) consist of Census villages in the rural sector and urban frame survey (UFS) blocks in the urban sector. The villages in which the survey is to be undertaken are selected by a probability proportional to size with replacement method, from the census listing of villages (NSSO, 2001).

The urban FSUs or UFS blocks are ‘mapped’ by the NSSO, taking into consideration the increase or decrease in the population of urban agglomerations and also newly declared towns, having clear identifiable boundaries and landmarks. Each block includes a population of 600 to 800 people living in approximately 200 households. Every year 20% of urban agglomerations are updated and over a period of 5 years, which is known as a ‘phase’, all blocks nationwide are updated (NSSO, 2001). The use of up-to-date maps is therefore essential when replicating the NSS methodology for research.

## Second Stage Sampling

### Selection of hamlet-groups and urban sub-blocks

The second stage sampling is done in the field since no list of households is available. First, large villages are divided into hamlet-groups and large blocks into sub-blocks. The number of these sub divisions depends on the population of the FSU. The steps followed to delineate these are:

- Step 1: The approximate population of the village is ascertained by seeking the advice of the Panchayat (village council), government school head-teacher or zilla parishad (local government office).
- Step 2: The community groupings or ‘hamlets’ are identified and their geographic boundaries are mapped.
- Step 3: The hamlets are grouped into NSSO ‘hamlet-groups’ for all villages with a population greater than 1200 (Table below).

Criteria applied for dividing the village into hamlet-groups

| Population | Number of hamlet-group/sub-blocks formed |
| --- | --- |
| Less than 1200 | No hamlet group/sub-blocks will be formed |
| 1200 to 1799 | 3 hamlet-group/sub-blocks will be formed |
| 1800 to 2399 | 4 hamlet-group/sub-blocks will be formed |
| 2400 to 2999 | 5 hamlet-group/sub-blocks will be formed |
| 3000 to 3599 | 6 hamlet-group/sub-blocks will be formed |

Source: (NSSO, 2004).

- Step 4: Two hamlet groups are randomly selected from these using simple random sampling without replacement and the listing of households is undertaken in these hamlet-groups.

For urban areas the identification of sub-blocks follows roughly similar steps but the sub-blocks are made up of approximately 200 households. Two sub-blocks are selected using simple random sampling without replacement for the purpose of the NSS (NSSO, 2004).

### Selection of households

To select the survey households, i.e. the second stage strata (SSS) or Ultimate Sampling Units (USU), households in the selected hamlet-groups and sub-blocks are listed. In our survey the following criteria were used to select 10 households just as NSSO does in the healthcare and morbidity round (Table below).

Criteria of selection of households in healthcare and morbidity round of NSSO

| **Composition of sub strata** | **With hamlet/group.sub block formation (for each)** |
| --- | --- |
| SSS 1: households with at least one member hospitalized during last 12 months | 4 |
| SSS 2: from the remaining households, households having at least one child of age below 5 years | 2 |
| SSS 3: from the remaining households, households with at least one member of age 60 years or above | 2 |
| SSS 4: other households | 2 |
| Sub total | 10 |

Source: (NSSO, 2004)
