# Supplementary material for: Private sector participation in delivering tertiary health care: a dichotomy of access and affordability across two Indian states
Source: Health Policy Plan. 2015 Mar 9;30(Suppl 1):i23–31. doi: 10.1093/heapol/czu061 (PMC4353890; doi:10.1093/heapol/czu061)
Supplement: Supplementary Data [file supp_czu061_Fig_1.docx]

**Fig 1 – The number of private hospitals receiving recognition as teaching hospitals per year by the Department of Medical Education in AP (APDME, 2012)**
