# Supplementary material for: Private sector participation in delivering tertiary health care: a dichotomy of access and affordability across two Indian states
Source: Health Policy Plan. 2015 Mar 9;30(Suppl 1):i23–31. doi: 10.1093/heapol/czu061 (PMC4353890; doi:10.1093/heapol/czu061)
Supplement: Supplementary Data [file supp_czu061_Table_1.docx]

**Table 1: Urban and rural populations and households surveyed in 2004 and 2012 in Andhra Pradesh and Maharashtra**

|  | **Andhra Pradesh** | | **Maharashtra** | |
| --- | --- | --- | --- | --- |
|  | **2004** | **2012** | **2004** | **2012** |
| Population in the entire state | 76,210,007* | 84,665,533** | 96,878,627* | 112,372,972** |
| Urban population in the state | 20,808,940* | 28,353,745** | 41,100,980* | 50,827,531** |
| Rural population in the state | 55,401,067* | 56,311,788 ** | 55,777,647* | 61,545,441** |
| Total households in the state (urban) | 4,397,138* | 6,778,225** | 8,403,224* | 10,813,928** |
| Total households in the state (rural) | 12,607,167* | 14,246,309** | 11,173,512* | 13,016,652** |
| Total households in the state | 17,004,305* | 21,024,534** | 19,576,736* | 23,830,580** |
| FSUs surveyed (urban) | 183^ | 372^^ | 267^ | 504^^ |
| FSUs surveyed (rural) | 325^ | 491^^ | 265^ | 504^^ |
| Total households surveyed (urban) | 1824^ | 3715^^ | 2664^ | 5038^^ |
| Total households surveyed (rural) | 3235^ | 4908^^ | 2650^ | 5035^^ |

*2001 census ** 2011 census ^ (NSSO, 2004)

FSU - First Stage Unit

^^The NSSO 66th round had 492 rural FSUs in AP, but 1 FSU was found to be uninhabited. The list of FSUs which were surveyed in the 66^th^ round were obtained from the Coordination and Publication Division of the National Survey Sample Organization after the investigators requested Deputy Director General to instruct their regional offices to provide these.
