# Supplementary material for: Private sector participation in delivering tertiary health care: a dichotomy of access and affordability across two Indian states
Source: Health Policy Plan. 2015 Mar 9;30(Suppl 1):i23–31. doi: 10.1093/heapol/czu061 (PMC4353890; doi:10.1093/heapol/czu061)
Supplement: Supplementary Data [file supp_czu061_Table_2.docx]

Table 2 Change in proportion of inpatient cases in public and private hospitals (among those hospitalized)

|  | In-patient cases | Baseline Mean (95% CI) | | Change 2004:2012 Mean (95% CI) | | DID Estimate | |
| --- | --- | --- | --- | --- | --- | --- | --- |
|  |  | Maharashtra | Andhra Pradesh | Maharashtra | Andhra Pradesh | Mean (95%CI) | P |
| Private | Overall | 0.72(0.7:0.73) | 0.7(0.69:0.72) | -0.011( -0.053:0.031) | 0.065(0.018:0.11) | 0.076(-0.012:0.14) | 0.02 |
|  |  | | | | | DID Estimate with covariates | |
|  |  |  |  |  |  | Mean (95%CI) | P |
|  |  |  |  |  |  | 0.05( -0.007:0.11) | 0.03 |
|  | Rural | 0.72(0.69:0.75) | 0.73(0.7:0.75) | 0.030(-0.027:0.089) | 0.028( -.023:0.081) | -0.0019( -0.080:0.076) | 0.96 |
|  | Urban | 0.72(0.70:0.75) | 0.63(0.6:0.66) | -0.067(-0.13: -0.0063) | 0.14(0 .047:0.23) | 0.21(0.095:0.31) | 0.0002 |
|  | Cardiac | 0.056(0.037:0.076) | 0.072(0.038:0.1) | -0.017( -.038:0.0038) | -0.018( -0.05:0.015) | -0.0015( -0.042:0.039) | 0.94 |
|  | Cardiac rural | 0.037(0.017:0.057) | 0.06(0.016-0.1) | -0.0056(-0.028:0.016) | -0.008(-0.055:0.037) | -0.0031( -0.054:0.048) | 0.9 |
|  | Cardiac urban | 0.08(0.046:0.011) | 0.097(0.057:0.13) | -0.028( -0.06:0.0085) | -0.04(-0.083:0.002) | -0.012(-0.069:0.043) | 0.65 |
|  | Nephrology | 0.035(0.047:0.09) | 0.069(0.047:0.09) | -0.027( -0.051:-0.004) | 0.0023(-.012:0.021) | 0.029(-.0036:0.06) | 0.053 |
|  | Nephrology- rural | 0.052(0.023:0.08) | 0.042(0.019:0.065) | -0.009( -0.026:0.02) | -0.006(-0.032:0.02) | 0 .0036(-0.037:0.04) | 0.86 |
|  | Nephrology urban | 0.088(0.055:0.12) | 0.018(0.005:0.031) | -0.04(-0.084:0.014) | 0.021(0.0009:0.041) | 0.07(0.03:0.11) | 0.0007 |
| Public | Overall | 0.27(0.23:0.31) | 0.3(0.26:0.34) | 0.011(-0.032:0.053) | -0.064(-0.11: -0.017) | -0.075(-0.14:0.0125) | 0.019 |
|  |  | | | | | DID Estimate with covariates | |
|  |  |  |  |  |  | Mean (95%CI) | P |
|  |  |  |  |  |  | -0.06( -0.11:0.005) | 0.074 |
|  | Rural | 0.28(0.21:0.35) | 0.27(0.23:0.31) | -0.03(-0.09:0.028) | -0.028(-0.08:0.02) | 0.0019( -0.076:0.08) | 0.96 |
|  | Urban | 0.26(0.21:0.32) | 0.36(0.28:0.45) | 0.067(-.062:0.12) | -0.14(-0.23:-0.047) | -0.2(-0.31:-0.095) | 0.0002 |
|  | Cardiac | 0.0034(0.02:0.049) | 0.045(0.025:0.065) | 0.005( -0.015:0.025) | -0.014(-0.038:0.11) | -0.019(-0.05:0.013) | 0.25 |
|  | Cardiac rural | 0.005(-0.00034:0.011) | 0.042(0.014:0.07) | -0.014( -0.012:0.04) | -0.021(-0.053:0.0098) | -0.036(-0.076: 0.00513) | 0.089 |
|  | Cardiac urban | 0.053(0.026:0.078) | 0.05(0.02:0.0786) | -0.008(-0.04:0.024) | 0.011(-0.031:0.054) | 0.019(-0.034:0.072) | 0.48 |
|  | Nephrology | 0.048(0.024:0.070) | 0.039( 0.0083:0.069) | -0.026(-0.05:-0.0016) | -0.012( -0.046:0.02) | 0.014( -0.028:0.055) | 0.52 |
|  | Nephrology- rural | 0.03(0.012:0.049) | 0.0078(0.0017:0.0014) | -0.014( -0.035:0.0076) | 0.018(-0.0029:0.036) | 0.031( 0.0034:0.059) | 0.028 |
|  | Nephrology urban | 0.069(0.023:0.11) | 0.083(0.011 :0.16) | -0.043( -0.09:0.0042) | -0.055(-0.13: -0.019) | -0.012(-0.1 :0.077) | 0.79 |
