# Supplementary material for: Private sector participation in delivering tertiary health care: a dichotomy of access and affordability across two Indian states
Source: Health Policy Plan. 2015 Mar 9;30(Suppl 1):i23–31. doi: 10.1093/heapol/czu061 (PMC4353890; doi:10.1093/heapol/czu061)
Supplement: Supplementary Data [file supp_czu061_Table_3.docx]

Table 3 Average expenditure among those hospitalized in the state (all values in INR deflated to 2004)

|  | In-patient cases | Baseline Mean (95% CI) | | Change 2004:2012 Mean (95% CI) | | DID Estimate | |
| --- | --- | --- | --- | --- | --- | --- | --- |
|  |  | Maharashtra | Andhra Pradesh | Maharashtra | Andhra Pradesh | Mean (95%CI) | P |
| Private | Overall | 5718.1(5118.6:6317.6) | 5758.8(4193.8:7323.9) | 3435.5(2521.7:4349.3) | 1358.9( -330.1:3048) | -2076.5( -3996:-157) | 0.04 |
|  |  | | | | | DID Estimate with covariates | |
|  |  |  |  |  |  | Mean (95%CI) | P |
|  |  |  |  |  |  | -2306.9( -4203:-410.7) | 0.017 |
|  | Rural | 6274.3(5547.4:7001.2) | 6545.2( 5766.6:7323.9) | 3397.9(2096.8:4699) | 1271.4(108.8:2434.024 | -1620.6( -3052: -189.3) | 0.026 |
|  | Urban | 9554.7(8059.4:11050) | 11804.8(5791.04:17818.5) | 2892.1(832.9:4951.5) | -2902(-9118.2:314.2) | -3235.3( -8006.4:1535.9) | 0.18 |
|  | Cardiac | 1065.2(618.3:1512.1) | 1067.6(617.2:1518) | 143.3( -472.8:759.3) | -228.7( -737.2:279.8) | -371.9( -1170.4:426.5) | 0.36 |
|  | Cardiac rural | 227.1(109:345.3) | 895.9(419.3:1372.6) | 337.7(-51.0444 726.5317 | -206.0( -747.0403 335.0375 | -543.7(-1209.6:122.2) | 0.11 |
|  | Cardiac urban | 2076.8(1107.2:3046.3) | 1453.9( 472.5106 2435.371 | 147.6(-1179.4:1474.7) | -259.5( -1370.6:851.6) | -407.1(-2137.1:1322.9) | 0.64 |
|  | Nephrology | 814.1(542.2:1086.01) | 292.3(155.8:428.9) | -220.1(-550.03:109.9) | 413.4( -8.2:835.1) | 633.5(98.3:1168.67) | 0.19 |
|  | Nephrology- rural | 479.3(248.5:710.1) | 364 183.085 544.82 | 77.09(-263.5:417.7) | 383.31(-194.1:960.7) | 306.2(-363.8:976.2) | 0.37 |
|  | Nephrology urban | 1217.7(685.1:1750.3) | 131.2(-43.8:306.3) | -564.2(-1165.7:37.4) | 475.5(101.8:849.2) | 1039.7(331.8:1747.6) | 0.004 |
| Public | Overall | 1440.4(1096:1785.005) | 1010.1(772.3:1247.9) | 7713.1(6944.7:8481.4) | 6107.7(5431.3: 6784.2) | -1605.3(-2628.6:-582.1) | 0.002 |
|  |  | | | | | DID Estimate with covariates | |
|  |  |  |  |  |  | Mean (95%CI) | P |
|  |  |  |  |  |  | -1711( -2776.1: -647.6) | 0.002 |
|  | Rural | 2223.5(1172.1:3274.8) | 1936(1460:2412.0) | 1897.1(241.3:3553) | 2212(677.5:3746.5) | -833.7(-2100.8: 433.5) | 0.2 |
|  | Urban | 2162.7(1449.0: 2876.4) | 1359.9( 855.9:1863.9) | 3957.4(2428.6:5486.2 | 1697.3(780.1:2614.5) | -2585.4(-4433.9:-736.9) | 0.0061 |
|  | Cardiac | 315.7(57:574.5) | 201.8( 23.9:379.6) | 333.5(-235.7:902.6) | -18.8( -226.1:188.6 | -352.2(-957.7253.2777 | 0.2541 |
|  | Cardiac rural | 331.4(-80.7:743.1) | 211.5(-66.3:489.3) | 209.5(-733.7:1152.7) | -70.6(-367:225.7009) | -384.5(-868.0:99) | 0.12 |
|  | Cardiac urban | 295.7(29.92:561.4) | 187.6(21: 354.2) | 467.6( -125.3:1060.4) | 106.9(-218..9:432.7) | -360.6(-1036.6:315.4) | 0.2956 |
|  | Nephrology | 84.7(7.5:161.9) | 104.2(50.5:158) | 55.0(-76.4:186.4) | 221.3(-120.4:563.1) | -166.3( -532.3:199.7) | 0.37 |
|  | Nephrology- rural | 58.4(2.4:114.4) | 24.7( -1.6:51.0) | 22.3(-66.5:111.0) | 110.8(-30.2:251.9) | 88.6(-78:55.1) | 0.3 |
|  | Nephrology urban | 163.1(65.1:261.1) | 171.7(-10.4:353.9) | 418.2( -276.1:1112.5) | -20.8(-243.4:201.8) | -439.0(-1167.7:289.6) | 0 .24 |
