# Supplementary material for: Private sector participation in delivering tertiary health care: a dichotomy of access and affordability across two Indian states
Source: Health Policy Plan. 2015 Mar 9;30(Suppl 1):i23–31. doi: 10.1093/heapol/czu061 (PMC4353890; doi:10.1093/heapol/czu061)
Supplement: Supplementary Data [file supp_czu061_Table_4.docx]

|  | | Baseline Mean (95% CI) | | Change 2004:2012 Mean (95% CI) | | DID Estimate | |
| --- | --- | --- | --- | --- | --- | --- | --- |
|  |  | Maharashtra | Andhra Pradesh | Maharashtra | Andhra Pradesh | Mean (95%CI) | p |
| Private | Overall | 6.6(6.1:7.2) | 10(8.1:11.8) | 0.18(-0.46:0.81) | -3(-4.9:-1.2) | -3.2(-5.3-1.2) | 0.002 |
|  |  | | | | | DID Estimate with covariates | |
|  |  |  |  |  |  | Mean (95%CI) | P |
|  |  |  |  |  |  | -3.2(-5.4:-1.2) | 0.003 |
|  | Rural | 6.9(6.2:7.7) | 10.5(8.1:12.9) | -0.1( -1:0.79) | -3.8(-2.9: -0.96) | -3.7(-6.3:-1) | 0.007 |
|  | Urban | 6.2(5.4: 7) | 8.9(6.5:11.2) | 0.5(-4:1.4) | -1.3( -3.7:-1.2) | -1.8(-4.4:0.8) | 0.17 |
| Public | Overall | 9.7(7.4:11.9) | 11.5(10.1:12.9) | -2.5(-4.9:-0.05) | -4.5(-6.3:-2.6) | -2(-5.1:1.1) | 0.2 |
|  |  | | | | | DID Estimate with covariates | |
|  |  |  |  |  |  | Mean (95%CI) | P |
|  |  |  |  |  |  | -2(-5.0:1.1) | 0.2 |
|  | Rural | 10.1(6.4:14) | 13.3(11.2:15.3) | -1.9(-6:2.1) | -6(-8.7:-3.5) | -4.2( -9:0.6) | 0.09 |
|  | Urban | 9.3(7.5:11) | 8.9(7.7:10) | -3(-4.9-:0.9) | -2(-3.8:-0.5) | 0.7( -1.8: 3.2) | 0.59 |

Table 4– The duration of hospital stay in days
